# Adaptation of glucocerebrosidase-producing CHO cells to serum-free suspension culture

**DOI:** 10.1186/1753-6561-8-S4-P46

**Published:** 2014-10-01

**Authors:** Juliana Branco Novo, Roselaine Campos Targino Valota, Ana Maria Moro, Isaias Raw, Paulo Lee Ho

**Affiliations:** 1 Centro de Biotecnologia, Instituto Butantan, São Paulo, SP, Brazil; 2 Laboratório de Biofármacos em Células Animais, Instituto Butantan, São Paulo, SP, Brazil

## Background

Deficiency of the lysosomal glucocerebrosidase (GCR) enzyme results in Gaucher disease, the most common inherited storage disorder [1]. Current treatment consists of enzyme replacement therapy by administration of exogenous GCR. Although effective, it is exceptionally expensive [2]. In Brazil, the public healthcare system provides the drug free of charge for all Gaucher's patients, which reaches the order of $ 84 million per year. However, the production of GCR by public institutions in Brazil would reduce significantly the therapy costs. With this purpose, we have previously developed a cell line producing recombinant GCR using anchorage-dependent CHO cells [3]. Recent advances in cell culture technology have allowed the elimination of serum along with the growth of cells in suspension mode. This mode is preferred in industrial production due to the well-understood principles of scaling parameters and the ease of process control. Additionally, the serum is expensive and a source of contaminants [4]. Here, we describe the adaptation of a CHO cell line producing human GCR to suspension culture in serum-free medium, and evaluate its effects on protein expression, glycosylation and enzymatic activity.

## Methods

Anchorage-dependent CHO cells producing recombinant human GCR in alpha-MEM supplemented with 10% serum were submitted to the process of adaptation to suspension culture in the chemically defined serum-free medium CD OptiCHO (Invitrogen). Two different strategies were used: direct adaptation (complete serum withdrawal in one passage) and sequential adaptation (reduction in serum concentration through several cell passages) in T-25 and T-75 culture flasks (not treated to avoid cell attachment), and 125 mL erlenmeyer flasks. Culture supernatants from adapted CHO cell clones were analyzed for GCR expression, using a murine anti-human GCR polyclonal antibody [5]. Glycosylation analysis of secreted GCR was performed by digestion with the endoglycosidases PNGase F and Endo H, and enzymatic activity was determined fluorometrically with the synthetic substrate 4-MUG.

## Results and conclusions

Although being laborious and very time-consuming, the process of adapting CHO cells producing recombinant GCR to growth in suspension culture and serum-free medium was successful. Protein bands of 66-69 kDa corresponding to secreted and glycosylated GCR were detected by western blotting analysis. Surprisingly, direct adaptation was more effective than sequential adaptation in obtaining high-producing clones. However, an unexpected decreased in the GCR expression was observed in long-term culture (40 days), indicating the presence of unstable cells producing GCR. In addition, the secreted protein was not active in the serum-free suspension culture, although glycosylation analysis showed a properly glycosylated GCR containing complex- and hybrid-type oligosaccharides, consistent with a functional enzyme. These results suggest that the stable production of an active GCR by CHO cells may be serum-dependent. The next steps include the addition of serum in the cell culture adapted to suspension. To our knowledge, this is the first report describing an adaptation process of CHO cells producing GCR for serum-free suspension culture
